# Skeletal muscle overexpression of sAnk1.5 in transgenic mice does not predispose to type 2 diabetes

**DOI:** 10.1038/s41598-023-35393-0

**Published:** 2023-05-20

**Authors:** E. Pierantozzi, L. Raucci, S. Buonocore, E. M. Rubino, Q. Ding, A. Laurino, F. Fiore, M. Soldaini, J. Chen, D. Rossi, P. Vangheluwe, H. Chen, V. Sorrentino

**Affiliations:** 1 Department of Molecular and Developmental Medicine, University of Siena, 53100 Siena, Italy; 2 Department of Cardio-Thoracic Surgery, Nanjing Drum Tower Hospital, Nanjing University Medical School, Nanjing, 210008 Jiangsu China; 3 Interdepartmental Program of Molecular Diagnosis and Pathogenetic Mechanisms of Rare Genetic Diseases, Azienda Ospedaliera Universitaria Senese, 53100 Siena, Italy; 4 Laboratory of Cellular Transport Systems, Department of Cellular and Molecular Medicine, Katholieke Universiteit Leuven (KU Leuven), 3000 Leuven, Belgium; 5 Programme in Cardiovascular and Metabolic Disorders, Duke-NUS Medical School, 8 College Road, Singapore, 169857 Singapore

**Keywords:** Genetics, Molecular biology

## Abstract

Genome-wide association studies (GWAS) and *cis-*expression quantitative trait locus* (cis-eQTL*) analyses indicated an association of the rs508419 single nucleotide polymorphism (SNP) with type 2 diabetes (T2D). rs508419 is localized in the muscle-specific internal promoter (P2) of the *ANK1* gene, which drives the expression of the sAnk1.5 isoform. Functional studies showed that the rs508419 C/C variant results in increased transcriptional activity of the P2 promoter, leading to higher levels of sAnk1.5 mRNA and protein in skeletal muscle biopsies of individuals carrying the C/C genotype. To investigate whether sAnk1.5 overexpression in skeletal muscle might predispose to T2D development, we generated transgenic mice (Tg^sAnk1.5/+^) in which the sAnk1.5 coding sequence was selectively overexpressed in skeletal muscle tissue. Tg^sAnk1.5/+^ mice expressed up to 50% as much sAnk1.5 protein as wild-type (WT) muscles, mirroring the difference reported between individuals with the C/C or T/T genotype at rs508419. However, fasting glucose levels, glucose tolerance, insulin levels and insulin response in Tg^sAnk1.5/+^ mice did not differ from those of age-matched WT mice monitored over a 12-month period. Even when fed a high-fat diet, Tg^sAnk1.5/+^ mice only presented increased caloric intake, but glucose disposal, insulin tolerance and weight gain were comparable to those of WT mice fed a similar diet. Altogether, these data indicate that sAnk1.5 overexpression in skeletal muscle does not predispose mice to T2D susceptibility.

## Introduction

Skeletal muscle tissue plays a key role in regulating overall body metabolism, including the systemic homeostasis of glucose. Indeed, accounting for approximately 50% of total body weight, it represents the largest insulin-sensitive tissue where a major part of glucose is disposed following physiological insulin stimulation^1,2^. Moreover, contraction, relaxation, storage of both amino acids and carbohydrates, and maintenance of body temperature are constitutive skeletal muscle physiological activities that require continuous ATP hydrolysis and regeneration^3,4^. This large-scale ATP/energy demand is substantially supported by glucose removal from the blood stream following the activation of insulin-dependent mechanisms^5^. Due to the considerable glucose demand of skeletal muscles, alterations in the complex mechanisms that regulate insulin-dependent glucose uptake and skeletal muscle metabolism may impact glucose homeostasis and predispose individuals to type 2 diabetes (T2D) development^6^.

T2D is the most common chronic metabolic disease, representing approximately 90% of the total cases of diabetes mellitus, a disease currently affecting approximately 500 million people worldwide, with a trend to significantly increase in the coming years^7^. T2D is characterized, in the early stages, by a reduced insulin response of peripheral tissues, particularly of skeletal muscle but also of the liver and adipose tissue^8–10^. Resistance to respond to insulin stimulation leads to a gradual increase in circulating insulin levels that, with time, may result in the exhaustion of pancreatic ß cell function^8,11^. T2D is a multifactorial disease whose development is influenced by both hereditary and environmental factors such as age, lifestyle, and diet^12^. In the last decade, genome-wide association studies (GWAS) have identified many loci in the human genome linked to T2D susceptibility, even though the functional interpretation of most of these loci remains challenging^12,13^. In several reports, genetic variants located within the *ANK1* gene have been associated with T2D^14–21^. The *ANK1* gene encodes Ankyrin1, a protein of approximately 200 kDa that operates as an adaptor that links integral plasma membrane proteins to the cortical cytoskeleton^22^. Although ubiquitously expressed, Ankyrin1 was initially identified as a component of the membrane-associated cytoskeleton that maintains the characteristic shape of erythrocytes and for the causative role of mutations in *ANK1* in hereditary spherocytosis^23^. In striated muscles, four tissue-specific small isoforms of Ankyrin1 (sAnk1s) with a molecular weight of approximately 20 kDa are expressed, namely, sAnk1.5, sAnk1.6, sAnk1.7, and sAnk1.9^24–27^. Among these four striated muscle-specific isoforms, sAnk1.5, a protein of only 17 kDa, is by far the most abundantly expressed^24^. sAnk1.5, in contrast to other larger Ankyrin1 isoforms^22^, contains a transmembrane segment that anchors this protein to the sarcoplasmic reticulum (SR) membrane, where it localizes to the M-band and, to a lesser extent, to the Z-disks of the sarcomere^24,27,28^. The cytoplasmic tail of sAnk1.5 contains a binding domain that allows the establishment of a direct interaction between sAnk1.5 and Obscurin, a giant sarcomeric protein^28–30^. The central role of sAnk1.5 and Obscurin in stabilizing the organization of the SR structure around myofibrils in skeletal muscle has been accurately delineated by several studies^27–33^. An additional potential role of sAnk1.5 in modulating the activity of the Sarco-Endoplasmic Reticulum Calcium ATPase (SERCA) pumps has also been proposed^34,35^.

More recently, a strong association between the rs508419 single nucleotide polymorphism (SNP), localized in the 3′ region of the ANK1 gene, and T2D susceptibility was reported^16,17,19^. In addition, an eQTL for the cis-acting regulation of sAnk1.5 expression in human skeletal muscle was identified for rs508419, and the increased expression of sAnk1.5 was associated with an increased risk of T2D^17^. Indeed, rs508419 resides in the alternative muscle-specific *ANK1* internal (P2) promoter that drives the expression of sAnk1.5 and is positioned in the intron between exons 39 and 40 in the 3′ region of the *ANK1* gene^25,26^. Experiments based on an in vitro luciferase assay performed on C2C12 muscle cells transfected with plasmids carrying the P2 promoter with either the C/C or T/T rs508419 variant demonstrated that the C/C variant presented increased transcriptional activity^16^. Accordingly, higher levels of sAnk1.5 mRNA and protein were found in skeletal muscle biopsies of individuals with the C/C genotype with respect to those with the T/T genotype^16,17^. Altogether, this evidence identifies the C/C variant of rs508419 as a risk allele for T2D susceptibility and suggests that increased expression of sAnk1.5 in skeletal muscle could represent a predisposing factor for T2D development^16–18^. To verify whether overexpression of sAnk1.5 in skeletal muscle tissue might be involved in predisposing to T2D susceptibility, we generated transgenic mice (Tg^sAnk1.5/+^) in which exogenous sAnk1.5 expression is under the transcriptional control of the muscle-specific rat myosin light chain (MLC) promoter^36^. Here, we describe the characterization of Tg^sAnk1.5/+^ mice and report the results of glucose and insulin tolerance evaluated in Tg^sAnk1.5/+ mice^ fed either chow or a high-fat diet. The results obtained indicate that overexpression of sAnk1.5 does not alter glucose homeostasis in Tg^sAnk1.5/+^ mice.

## Results

### sAnk1.5 expression pattern in Tg^sAnk1.5/+^ mice

**Figure 1:**
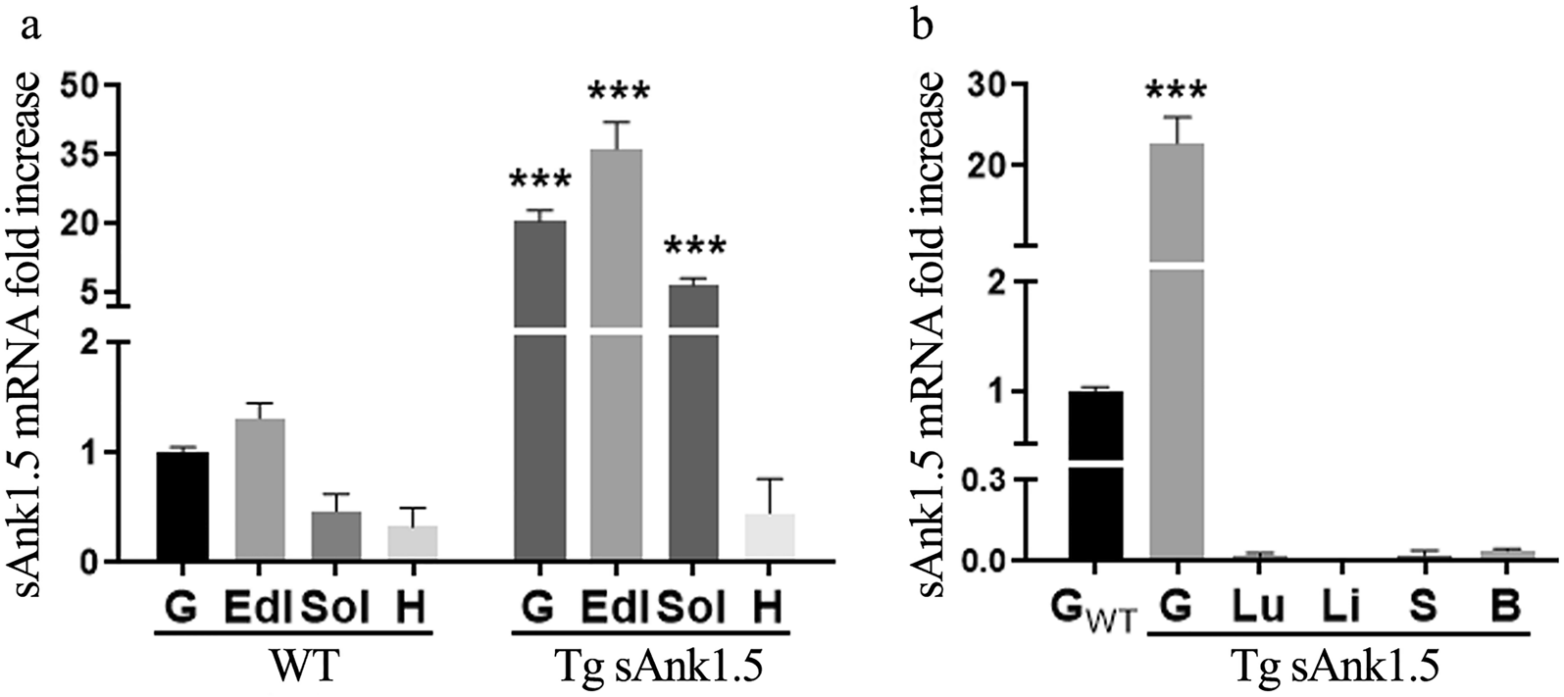
sAnk1.5 mRNA expression in Tg^sAnk1.5/+^. (**a**) Relative sAnk1.5 mRNA expression by quantitative RT-PCR analysis in different WT and transgenic striated muscles (gastrocnemius, G; extensor digitorum longus, EDL; soleus, Sol; heart, H). (**b**) Relative sAnk1.5 mRNA expression in different transgenic tissues (Lung, Lu; Liver, Li; Spleen, S; Brain, B). In both graphs, sAnk1.5 mRNA levels are reported as the fold increase ± SD relative to the reference. In (**a**) (for WT muscles) and (**b**), the mRNA level detected in WT gastrocnemius was chosen as a reference and arbitrarily set as “1”. The fold increase in sAnk1.5 mRNA levels in transgenic muscles reported in (**a**) was calculated using the corresponding WT muscle as a reference. ***p < 0.001, Student’s t-test.

Previous studies showed that the T2D-associated rs508419 C/C genotype increases the activity of the *ANK1* P2 promoter, leading to higher levels of sAnk1.5 mRNA and protein in skeletal muscle biopsies of individuals carrying the C/C genotype^16,17^. To investigate whether sAnk1.5 overexpression in skeletal muscle might result in predisposition to T2D, we subcloned the coding sequence of murine sAnk1.5 (GenBank accession number: U73972) into the pMEX expression vector, which carries the rat skeletal muscle MLC promoter and its enhancer and the SV40 poly-A sequence, thus generating the pMex-sAnk1.5 plasmid^36^. Microinjection of the pMex-sAnk1.5 DNA into the pronuclei of fertilized one-cell mouse embryos resulted in the generation of Tg^sAnk1.5/+^ mice. The Tg^sAnk1.5/+^ mouse colony was expanded in a hemizygous state. Tg^sAnk1.5/+^ mice were fertile, were born at the expected Mendelian ratio, and presented an indistinguishable phenotype from WT sibling mice. As shown in Fig. 1a, in the gastrocnemius, extensor digitorum longus (EDL), and soleus muscles of Tg^sAnk1.5/+^ mice, sAnk1.5 mRNA levels were increased by approximately 20-, 35- and 6-fold, respectively, compared to WT muscles. Conversely, we did not detect a significant increase in sAnk1.5 mRNA in the hearts of Tg^sAnk1.5/+^ mice with respect to WT animals (Fig. 1a). As expected, sAnk1.5 mRNA was almost undetectable in the other tissues of Tg^sAnk1.5/+^ mice (Fig. 1b).

**Figure 2:**
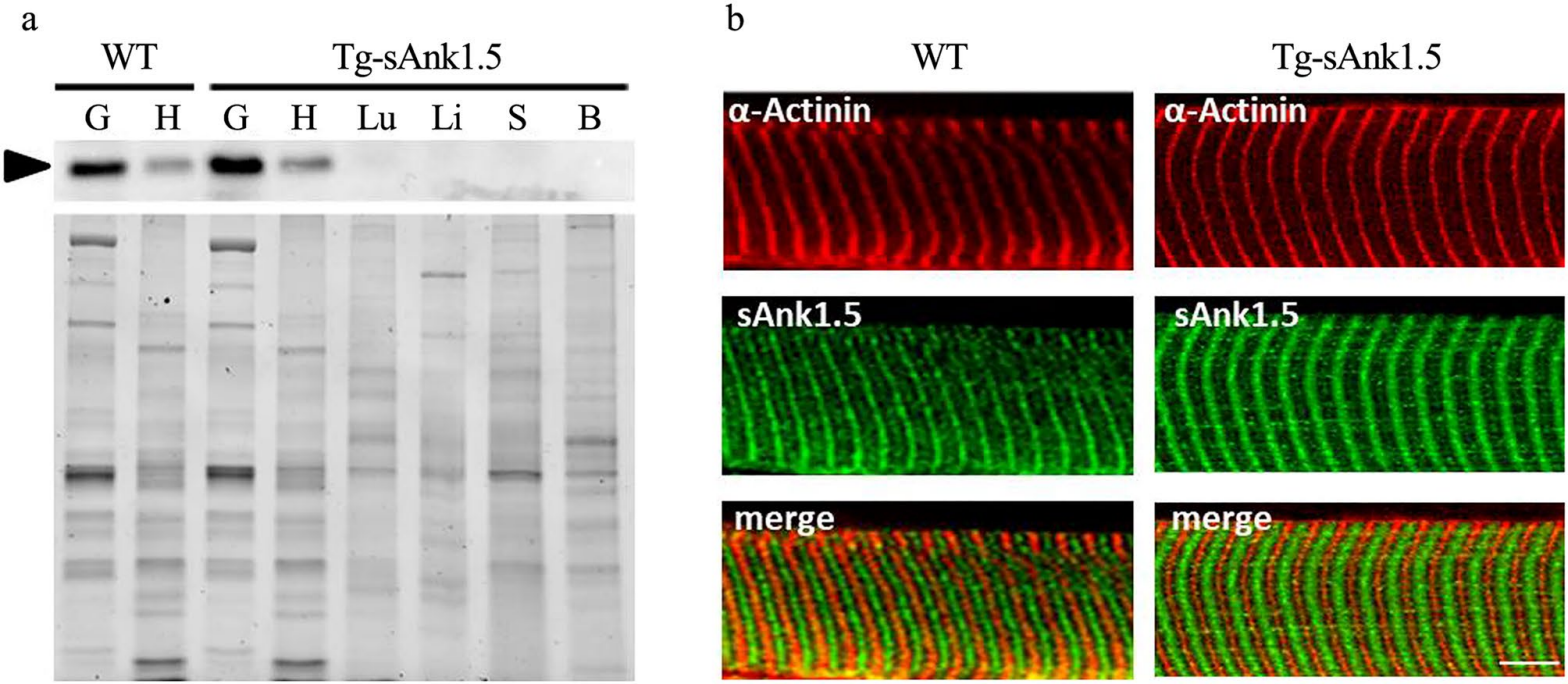
sAnk1.5 protein expression in Tg^sAnk1.5/+^. (**a**) Representative western blot analysis of sAnk1.5 (arrowhead) expression in different tissues of transgenic mice (Gastro, G; Heart, H Lung, Lu; Liver, Li; Spleen, S; Brain, B). Total loaded proteins were detected by Stain-Free technology (lower panel). (**b**) Immunofluorescent staining of sAnk1.5 (green) in EDL fibers isolated from WT and Tg^sAnk1.5/+^ mice. α-Actinin staining (red) was used to decorate the Z-disk. Fluorescent signal overlap is reported in the merge panel. Bar = 5 µm.

In agreement with the observed sAnk1.5 mRNA expression pattern, sAnk1.5 protein expression was exclusively confined to the skeletal and cardiac muscles of Tg^sAnk1.5/+^ mice (Fig. 2a). In addition, sAnk1.5 was localized in correspondence with the M-band and, to a lesser extent, the Z-disk of the sarcomere (Fig. 2b), completely mirroring the localization pattern of endogenous sAnk1.5 observed in WT fibers^24,28^.

**Figure 3:**
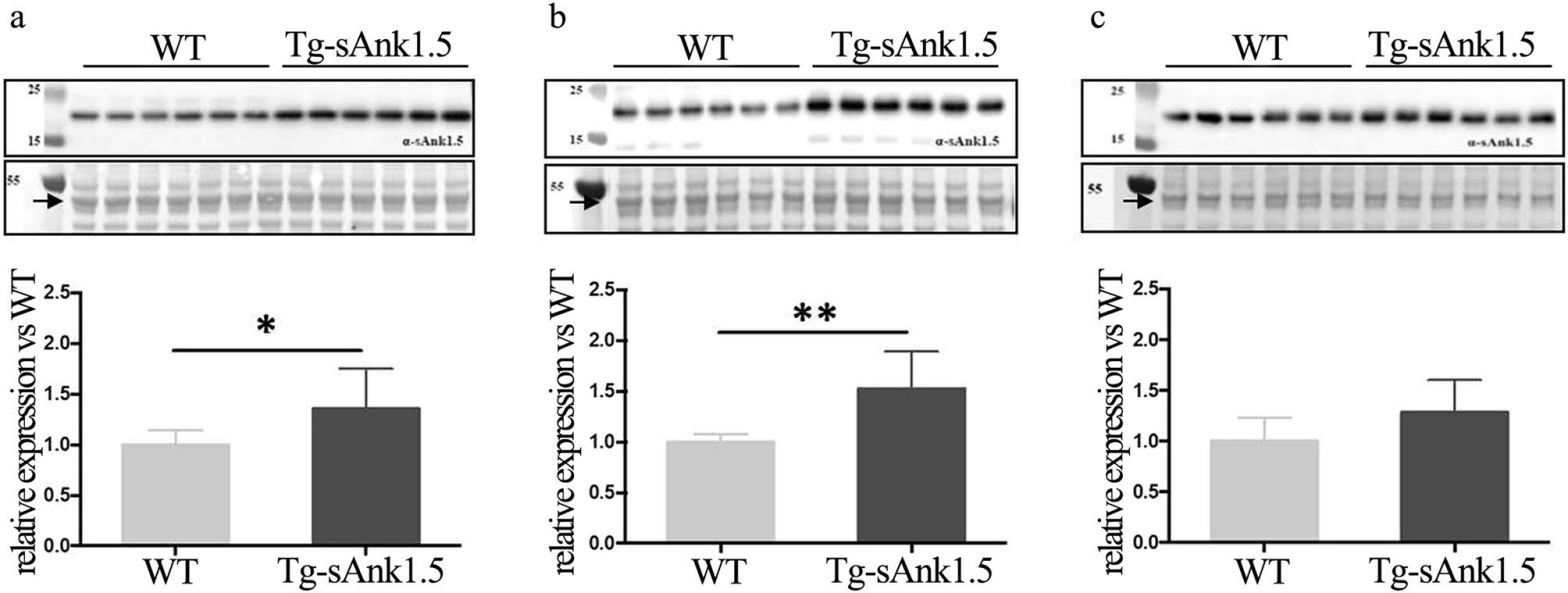
sAnk1.5 protein expression in the skeletal muscles of Tg^sAnk1.5/+^. Western blot analysis of sAnk1.5 protein expression in gastrocnemius (**a**), EDL (**b**), and soleus (**c**) muscles (n = 6 per mouse line, upper panels) isolated from 4-month-old mice. Densitometric analyses using actin (arrow in the ponceau S panels) as a normalizer are reported in the histograms in the bottom panels. sAnk1.5 protein levels in transgenic muscles are reported as fold increase ± SD relative to WT muscles. *p < 0.05, **p < 0.01, Student’s t-test. Original blots/gel are presented in Supplementary Fig. S1 online.

The analysis of sAnk1.5 expression in gastrocnemius, EDL and soleus muscles revealed an increase in sAnk1.5 protein levels in Tg^sAnk1.5/+^ gastrocnemius and EDL of 43% and 52% over WT muscles, respectively (Fig. 3a,b and Supplementary Fig. S1 online). A reproducible increase of approximately 25% in sAnk1.5 protein levels was observed in the soleus muscle of Tg^sAnk1.5/+^ mice, although it did not reach the level of statistical significance (p = 0.09) (Fig. 3c and Supplementary Fig. S1 online). The differences in sAnk1.5 expression levels among these different skeletal muscles are likely to reflect the preferential expression of the MLC promoter in glycolytic/fast twitch fibers, which are more abundant in gastrocnemius and EDL muscles, while the soleus muscle is enriched in oxidative/slow twitch fibers^36,37^.

### Glucose and insulin tolerance in Tg^sAnk1.5/+^ mice fed a standard diet

**Figure 4:**
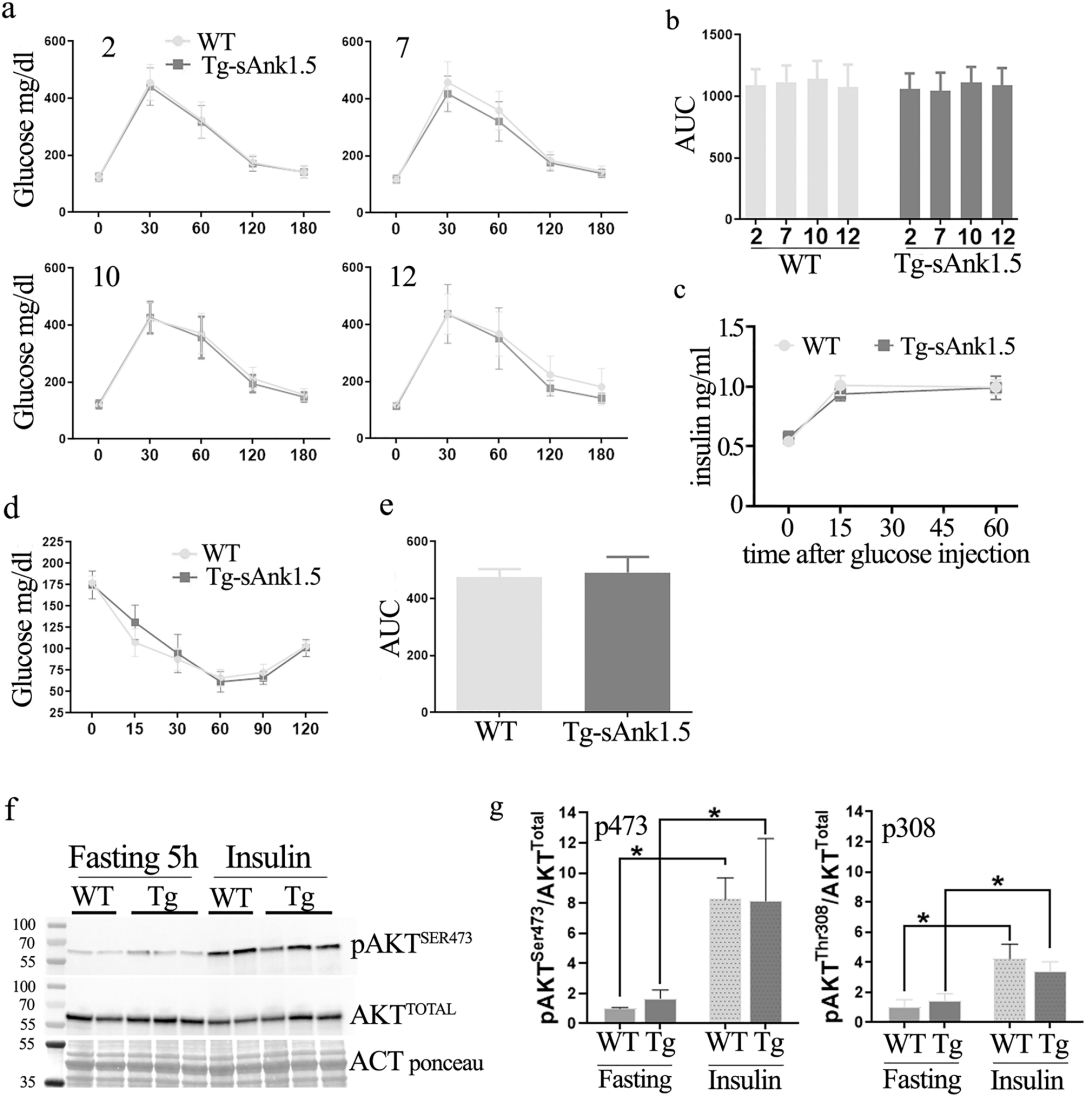
Intra-peritoneal glucose (IPGTT), insulin levels and intra-peritoneal insulin tolerance tests (IPITT) in chow-fed mice. (**a**) IPGTT was performed on WT and transgenic mice at 2, 7, 10 and 12 months of age (n = 25 WT and 25 Tg^sAnk1.5/+^; 22 WT and 24 Tg^sAnk1.5/+^; 25 WT and 25 Tg^sAnk1.5/+^; 15 WT and 16 Tg^sAnk1.5/+^, respectively). Mice were fasted overnight for 17 h, and glucose (2 g/kg) was administered through intraperitoneal injection. Blood glucose concentration was measured at 0, 30-, 60-, 120-, and 180-min following injection. (**b**) AUC ± SD calculated from glycemic curves reported in (**a**). (**c**) Serum insulin levels (ng/ml ± SD) in WT and Tg^sAnk1.5/+^ mice following 17 h of fasting, and 15 and 60 min after glucose administration (2 g/kg). (**d**) IPITT was performed on 12-month-old WT and transgenic mice. Mice were fasted daily for 5 h, and insulin (1 U/kg) was administered through intraperitoneal injection. Blood glucose concentration was measured at 0, 15-, 30-, 60-, 90-, and 120 min following injection. **e**: AUC ± SD calculated from glycemic curves reported in (**d**). (**f**) Western blot analyses of AKT phosphorylation at serine 473 in the gastrocnemius muscle from WT and Tg^sAnk1.5/+^ mice. (**g**) Densitometric analysis of p-AKT^Ser473^, p-AKT^Thr308^ (see also Supplementary Fig. S2 online) and total AKT, using actin (ponceau S staining) as a normalizer, is expressed as fold levels of pAKT/totalAKT ratio ± SD, relative to WT mice. *p < 0.01, Student’s t-test. Original blots/gels are reported in Supplementary Fig. S2 online.

To assess whether increased levels of sAnk1.5 would be responsible for the induction of a (pre-) diabetic phenotype, we monitored glucose tolerance in chow fed Tg^sAnk1.5/+^mice for a period of twelve months. Intraperitoneal glucose tolerance tests (IPGTTs) were performed by intraperitoneal injection of glucose (2 g/kg) in mice fasted overnight, and blood glucose concentrations were measured at different time points following injection. IPGTT performed at 2, 7, 10 and 12 months of age revealed no difference in blood glucose concentration between Tg^sAnk1.5/+^ and WT mice at any of the analyzed time points, including starting basal values after overnight fasting (Fig. 4a). Overall glucose tolerance, calculated as the area under the glycemic curve (AUC), was not altered in Tg^sAnk1.5/+^ mice with respect to age-matched WT mice (Fig. 4b). Serum insulin levels after intraperitoneal injection of glucose (2 g/kg) were also evaluated. As reported in Fig. 4c, the insulin levels in Tg^sAnk1.5/+^ and WT mice at 10 months of age were comparable after 17 h of fasting and at 15 and 60 min after glucose injection. We next tested the response to insulin in Tg^sAnk1.5/+^ mice at 12 months of age in a standard intraperitoneal insulin tolerance test (IPITT). As shown in Fig. 4d,e, again no difference was observed between Tg^sAnk1.5/+^ and WT mice. Finally, AKT phosphorylation levels on Ser473 and Thr308 were evaluated in Tg^sAnk1.5/+^ and WT mice after 5 h of fasting, and at 15 min after insulin injection. As reported in Fig. 4f,g and Supplementary Fig. S2 online, no difference in AKT phosphorylation levels was observed between Tg^sAnk1.5/+^ and WT mice in either condition.

### Glucose and insulin tolerance in Tg^sAnk1.5/+^ mice fed a high-fat diet

**Figure 5:**
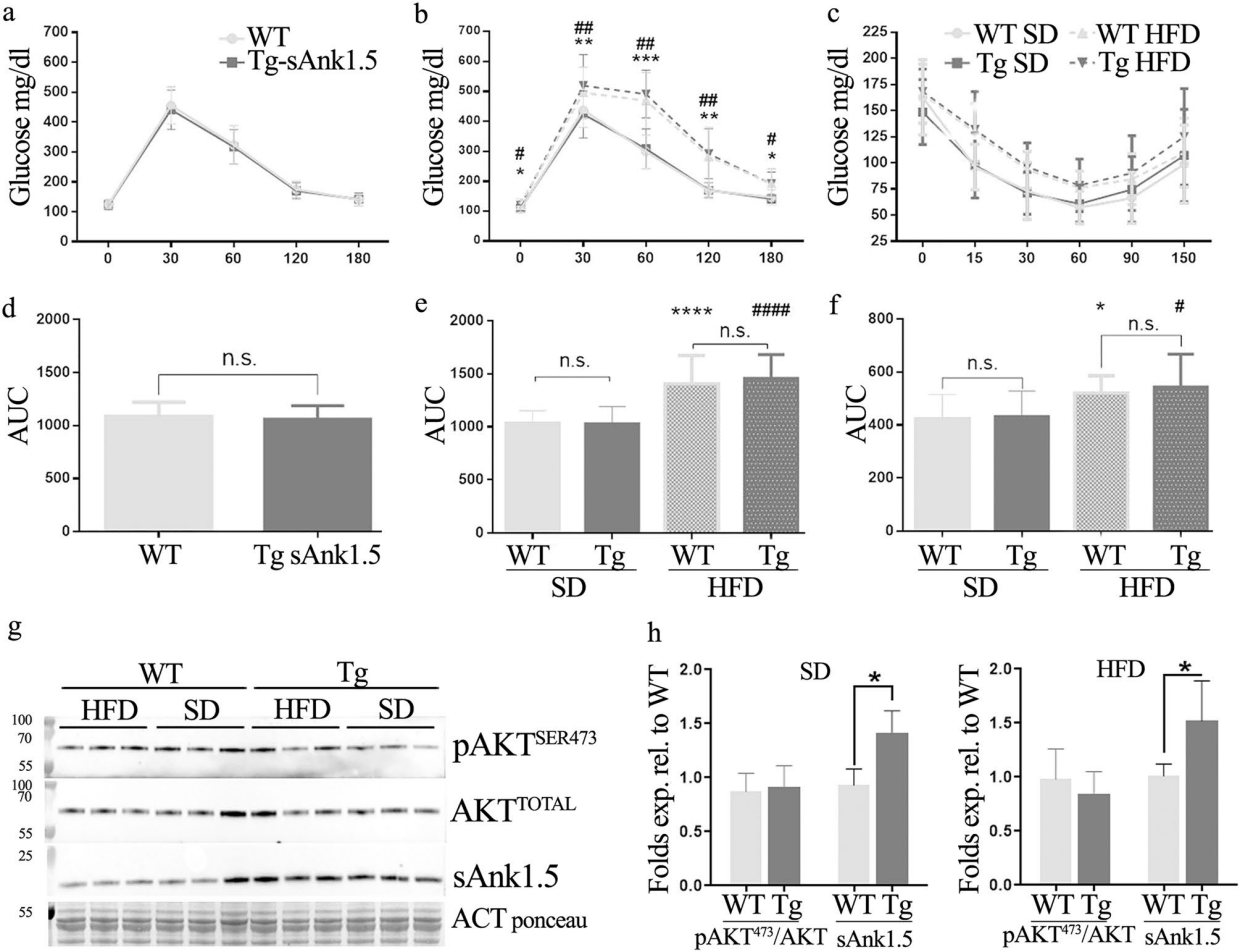
IPGTT and IPITT on high fat fed mice. 2-month-old mice were divided into four groups and fed either chow (standard diet; SD) or a high-fat diet (HFD) for twelve weeks (n = 18 SD WT, 18 SD Tg^sAnk1.5/+^, 18 HFD WT, 20 HFD Tg^sAnk1.5/+^). (**a**) IPGTT of 2-month-old WT and Tg^sAnk1.5/+^ mice before starting a high-fat diet (n = 36 WT and 38 Tg^sAnk1.5/+^). (**b**) IPGTT of WT and Tg^sAnk1.5/+^ mice at the end of the high-fat diet period. (**c**) IPITT of WT and Tg^sAnk1.5/+^ mice at the end of the high-fat diet period. (**d**) areas under the curves (AUC ± SD) calculated from glycemic curves at 2 months of age reported in (**a**). € AUC ± SD calculated from glycemic curves of chow (SD)- and high fat (HFD) fed mice at the end of the high-fat diet period reported in (**b**). (**f**) AUC ± SD calculated from the IPITT experiment reported in (**c**). * and ^#^ refer to WT SD vs WT HFD and Tg^sAnk1.5/+^ SD vs Tg^sAnk1.5/+^ HFD, respectively; * and ^#^, ** and ^##^, ***, **** and ^####^p < 0.05, < 0.01, < 0.001, < 0.0001, respectively; n.s. = not significant. (**g**) representative western blot analysis of AKT phosphorylation at serine 473 in the gastrocnemius of chow-fed (SD) and high-fat diet-fed (HFD) WT and Tg^sAnk1.5/+^ mice. (**h**) Densitometric analysis of p-AKT^Ser473^ and total AKT, using actin (ponceau S staining) as a normalizer, for both dietetic regimens. Data are expressed as pAKT^473^/total AKT ratio ± SD relative to WT diet-matched mice. Densitometric analysis of sAnk1.5 signal intensity relative to WT diet-matched mice is also reported. *p < 0.05, Student’s t-test. Original blots/gel are reported in Supplementary Fig. S3 online.

Since susceptibility to diabetes may not be disclosed with aging alone, high-fat diets are routinely used to induce obesity and to increase fat mass to hasten insulin resistance and worsen glucose tolerance in susceptible mice^38^. To understand whether sAnk1.5 overexpression might predispose to T2D susceptibility, 2-month-old WT and Tg^sAnk1.5/+^ mice were fed a high-fat diet for a period of twelve weeks. Control groups for both genotypes were fed a standard chow diet in parallel. In agreement with previous data (Fig. 4), the glucose tolerance of Tg^sAnk1.5/+^ and WT mice was similar before starting the high-fat diet protocol (Fig. 5a,d). As expected, 12 weeks of a high-fat diet reduced the levels of glucose tolerance and increased the levels of insulin resistance in both Tg^sAnk1.5/+^ and WT mice compared to chow-fed genotype-matched mice. However, even after undergoing a high-fat diet, the glucose tolerance and insulin response of Tg^sAnk1.5/+^ mice did not differ from those of diet-matched WT mice (Fig. 5b,c,e,f). Accordingly, phosphorylated AKT levels, which are usually impaired in (pre)diabetic mouse models^39–41^, were comparable in high-fat diet-fed Tg^sAnk1.5/+^ and WT mice (Fig. 5g,h and Supplementary Fig. S3 online).

**Figure 6:**
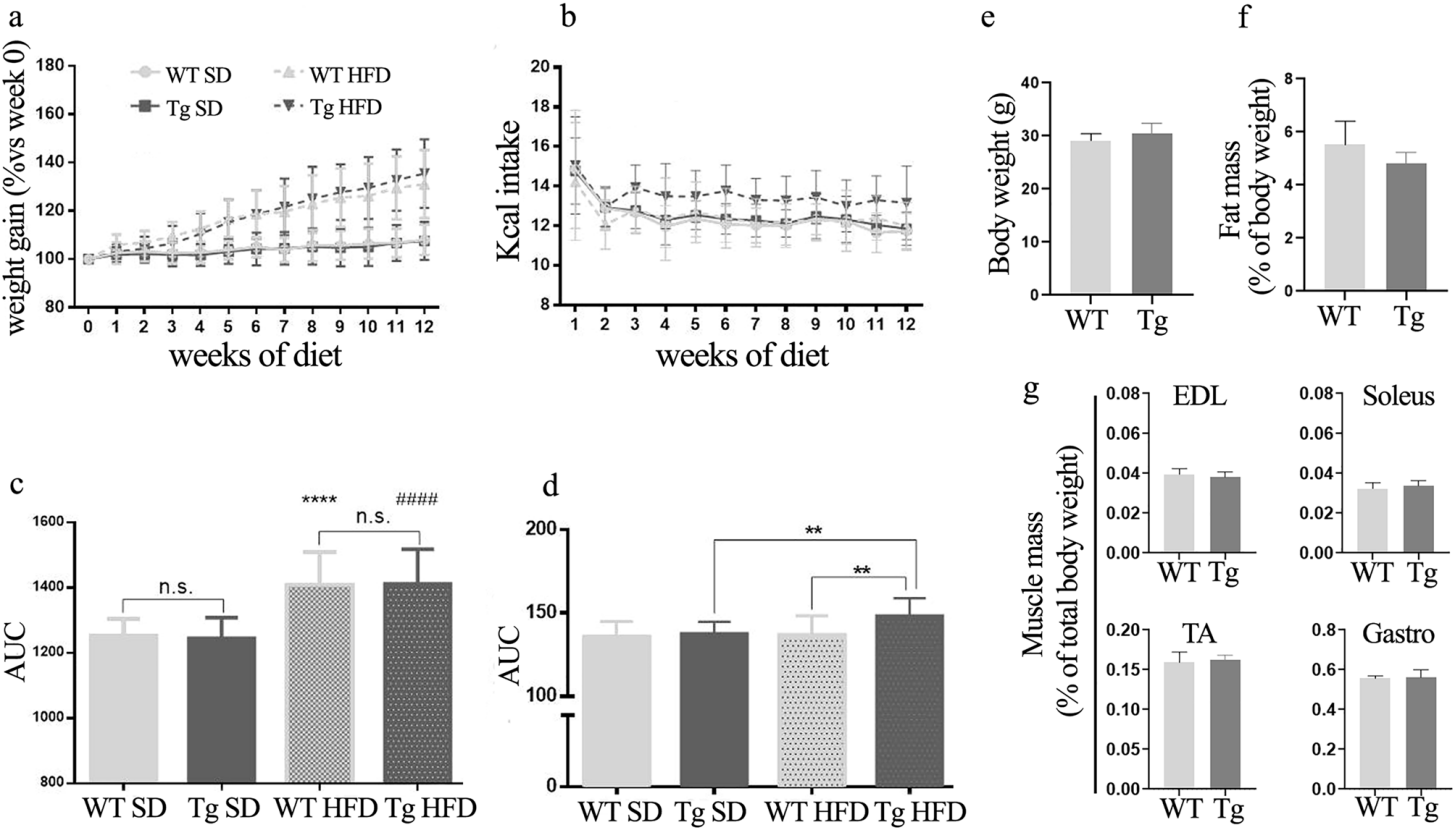
Weight gain and Kcal intake during the high-fat diet period. (**a**) Percentage of weekly body weight gain compared to week 0 (before starting HFD protocol). (**b**) Daily Kcal intake calculated from weekly grams of food intake. **c**: AUCs ± SD calculated from body weight gain graphs reported in (**a**). (**d**) AUCs ± SD calculated from Kcal intake graphs reported in (**b**); **p < 0.01; ****, ^####^p < 0.0001; n.s = not significant. (**e**) Body weight (grams ± SD) of chow fed 8-months-old mice. (**f**) Fat mass (total dissected adipose tissues, see also Supplementary Fig. S4 online) of 8-months-old mice reported as percentage ± SD of total body weight. (**g**) EDL, soleus, tibialis anterior (TA) and gastrocnemius (gastro) mass of 8-months-old mice reported as percentage ± SD of total body weight.

Calorie intake and body weight gain were also monitored during the 12 weeks of high-fat feeding. WT and Tg^sAnk1.5/+^ mice fed a chow diet gained weight at comparable rates (Fig. 6a,c,e) and displayed identical daily caloric intake (Fig. 6b,d). In addition, the amount of body fat and muscle mass was also quantified by dissecting adipose tissue (cervical, interscapular, axillo-thoracic, mesenteric, abdominal pelvic, retroperitoneal, epididymal and inguinal, see Supplementary Fig. S4 online) and EDL, soleus, tibialis anterior (TA) and gastrocnemius muscles from 8-months-old WT and Tg^sAnk1.5/+^ mice. As reported in Fig. 6f,g, no differences were observed between WT and Tg^sAnk1.5/+^ mice, either in terms of fat mass (WT = 1.58 g ± 0.10; Tg^sAnk1.5/+^  = 1.47 g ± 0.23), or in terms of skeletal muscle mass (EDL WT = 11.66 mg ± 1.07 and EDL Tg^sAnk1.5/+^  = 11.60 ± 1.09; Soleus WT = 9.57 mg ± 1.23 and Soleus Tg^sAnk1.5/+^  = 10.26 ± 1.14; TA WT = 46.85 mg ± 3.13 and TA Tg^sAnk1.5/+^  = 49.55 ± 5.07; Gastrocnemius WT = 163.92 mg ± 7.27 and Gastrocnemius Tg^sAnk1.5/+^ = 170.56 ± 13.74). Notably, Tg^sAnk1.5/+^ mice fed a high-fat diet displayed a significant increase in caloric intake compared to WT mice (Fig. 6b). Nonetheless, the weight gain of Tg^sAnk1.5/+^ and WT mice fed a high-fat diet was found to be similar (Fig. 6a).

### SERCA activity in Tg^sAnk1.5/+^ mice

**Figure 7:**
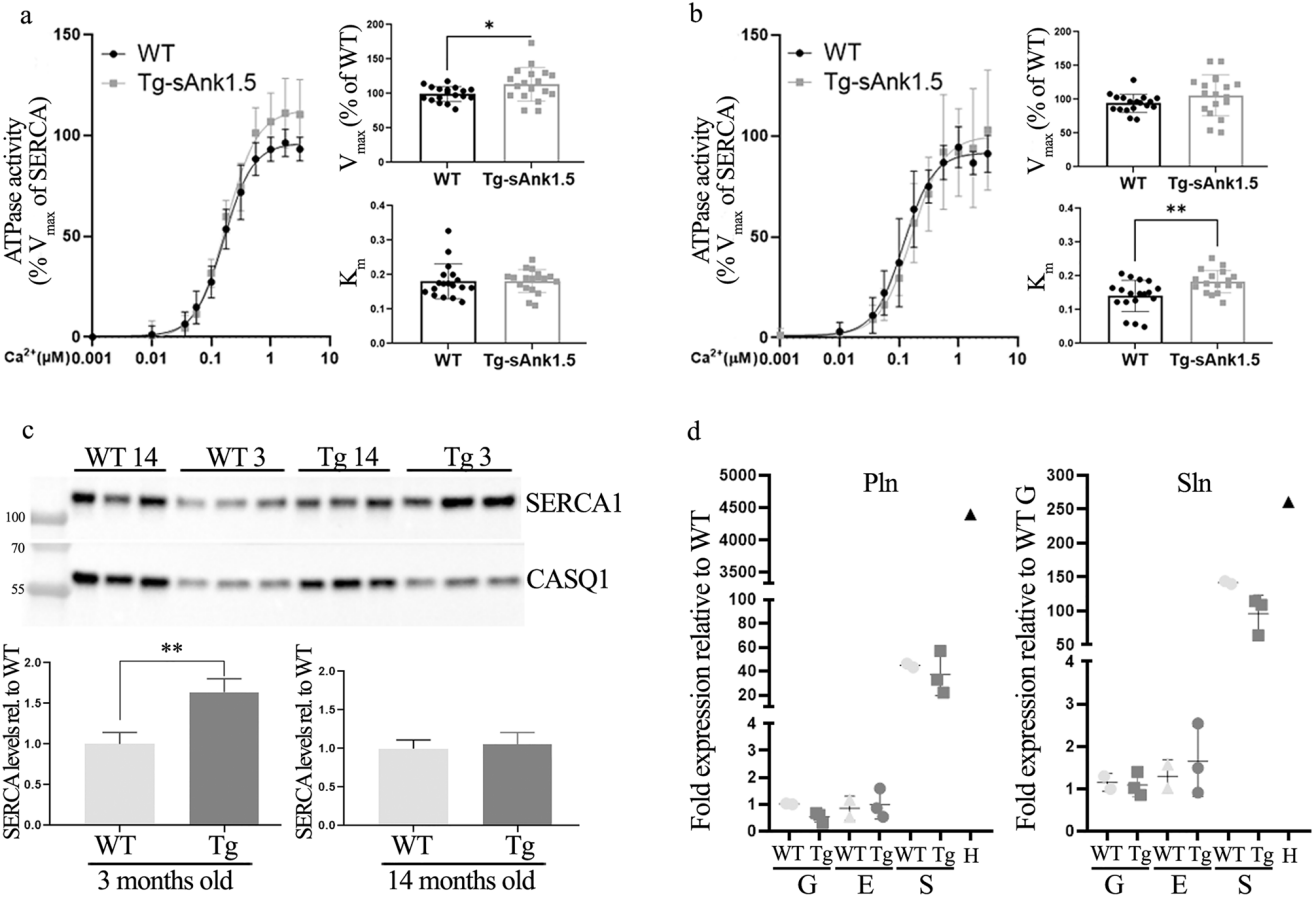
SERCA1 and p-AKT analysis. Ca^2+^-dependent ATPase activity of SERCA1a in 3-month-old (**a**) and 14-month-old (**b**) gastrocnemius muscles excised from WT and Tg^sAnk1.5/+^ mice. Specific ATPase activity as a function of [Ca^2+^] is reported as a percentage of WT V_max_. Error bars represent standard deviation. K_m_ and V_max_ of the enzymatic reaction are reported next to the enzymatic activity curves. (**c**) Representative western blot analyses of SERCA protein levels in microsomes prepared from the gastrocnemius of 3- and 14-month-old WT and Tg^sAnk1.5/+^. Densitometric analysis of SERCA signal intensity in 3- and 14-month-old mice was performed using Calsequestrin1 as a normalizer. p = * < 0.05, Student’s t-test. Original blots/gel are presented in Supplementary Fig. S5 online. (**d**) Relative phospholamban (Pln) and sarcolipin (Sln) mRNA expression by quantitative RT-PCR analysis in gastrocnemius (G), extensor digitorum longus (E) and soleus (S) muscles from WT and Tg^sAnk1.5/+^. Cardiac mRNA (H) was used as positive control. In both graphs, Pln and Sln mRNA levels are reported as fold increase ± SD relative to WT gastrocnemius.

The increased calorie intake that we observed in high-fat diet-fed Tg^sAnk1.5/+^ mice was suggestive of a potential increase in energy demand. Ca^2+^ reuptake in the SR by SERCA pump activity is a major ATP-consuming mechanism in skeletal muscle, accounting for almost half of the whole resting metabolic rate of this tissue^42^. Accordingly, SERCA1 activity was evaluated by Ca^2+^-dependent ATPase assay on microsome preparations obtained from the gastrocnemius muscle excised from 3- and 14-month-old WT and Tg^sAnk1.5/+^ mice. As shown in Fig. 7a, the V_max_ of the enzymatic reaction was significantly increased in 3-month-old Tg^sAnk1.5/+^ muscles compared to age-matched WT muscles, while K_m_ was unchanged between the two groups at 3 months of age. Conversely, the K_m_ of the enzymatic reaction was significantly increased in 14-month-old Tg^sAnk1.5/+^ muscles compared to age-matched WT muscles, while V_max_ values were comparable between the two groups at 14 months of age (Fig. 7b). Beyond sarcolipin and phospholamban, which are the most characterized SERCA pump regulators, in recent years, additional small molecular weight proteins, including sAnk1.5, have emerged as effective or potential regulators of SERCA activity^34,35,43–47^. Notably, a positive correlation between the mRNA levels of sAnk1.5 and SERCA1 has been previously described^16^ and altered SERCA expression levels were previously observed in a rat model of T2D^48^. To verify whether basal SERCA expression might be altered in skeletal muscles overexpressing sAnk1.5, we analyzed SERCA1 protein levels in the gastrocnemius muscle of sAnk1.5 transgenic mice at 3 and 14 months of age. As shown in Fig. 7c and Supplementary Fig. S5 online, SERCA1 protein levels were significantly increased in the muscles of 3-month-old Tg^sAnk1.5/+^ mice compared to WT mice of the same age, while they were comparable when analyzed in the muscles of 14-month-old mice. SERCA activity has been shown to be regulated by a number of proteins, with sarcolipin (Sln) and phospholamban (Pln) being the most studied modulators of SERCA activity^45,47,48^. Accordingly, protein levels of Sln and Pln were investigated by western blot technique. As shown in Supplementary Fig. S6 online, Pln levels were just about detectable, at similar low levels, in soleus muscle of both WT and Tg^sAnk1.5/+^ mice, but were not detectable in EDL and gastrocnemius muscles. In spite of our efforts, we were not able to obtain a reliable Sln signal by western blot in the skeletal muscles analyzed and in the heart tissue from both WT and Tg^sAnk1.5/+^ mice. Therefore, to better verify whether expression of Pln and Sln could differ between WT and Tg^sAnk1.5/+^ mice, qPCR experiments were performed. As shown in Fig. 7d, although the levels of expression of Pln and Sln differed between gastrocnemius, EDL and soleus muscles, no significant difference in the levels of Pln and Sln transcripts was observed between WT and Tg^sAnk1.5/+^ mice.

## Discussion

Skeletal muscle constitutes the largest insulin-sensitive tissue in the body, thus representing a major site for insulin-stimulated glucose utilization. As a crucial consumer of glucose, to supply the energy required for contraction, Ca^2+^ reuptake in the SR, and thermogenesis, skeletal muscle has a major contribution to both systemic glucose homeostasis and whole-body energy metabolism. Not surprisingly, the complex mechanisms that regulate insulin-dependent glucose uptake and metabolism in skeletal muscles have also been found, if altered, to play a critical role in the development of T2D, which in fact, in the early stages, is characterized by a reduced insulin response of peripheral tissues, especially skeletal muscle, but also liver and adipose tissue^1,6,49,50^.

T2D is a multifactorial metabolic disease in which known environmental risk factors, including poor diet, obesity, low levels of physical activity, and older age, can be accompanied and exacerbated by specific genetic variants/mutations, leading to increased susceptibility to T2D. Over the past years, several GWAS identified the *ANK1* gene as a common locus associated with T2D susceptibility^14–21,51–55^. However, none of the identified variants provided evidence for a potential mechanism by which the *ANK1* gene could contribute to T2D. More recent advances in understanding the potential role of the *ANK1* locus have been obtained by studies in which GWAS data were combined with the analysis of tissue-specific transcriptome data and analysis of chromatin accessibility^17,52–55^. These studies provided evidence that the rs508419 C/C variant is associated with T2D and that this genotype results in increased expression levels of sAnk1.5 mRNA and protein in skeletal muscle biopsies of individuals carrying the C/C genotype^16,17,19^.

Validation of the pathological role of a given potential candidate identified by observational studies of genome-disease association ultimately relies on biological assays and, whenever possible, on studies in transgenic models^56–59^. Accordingly, to determine the potential effects of increased sAnk1.5 in predisposing to T2D, we generated a transgenic mouse model, Tg^sAnk1.5^, in which the sAnk1.5 coding sequence was selectively overexpressed in striated muscle. Characterization of the Tg^sAnk1.5^ mice revealed a 6- to 35-fold increase in exogenous sAnk1.5 transcripts, with the highest levels in fast muscles and the lowest in slow muscles. This significant increase in sAnk1.5 mRNA, however, resulted in only a modest increase in sAnk1.5 protein levels, which were only increased by approximately 50% in the skeletal muscle of Tg^sAnk1.5/+^ mice compared to WT mice.

Differences between sAnk1.5 mRNA and protein levels have been previously reported and explained to be due to posttranslational control of the sAnk1.5 protein by a cullin-dependent mechanism of degradation^32,60^. Nevertheless, we observed that sAnk1.5 was correctly localized on the SR of Tg^sAnk1.5/+^ muscles, suggesting that transgenic fibers can adequately accommodate such an increase in sAnk1.5 protein.

However, overexpression of exogenous sAnk1.5 in Tg^sAnk1.5/+ mice^ did not alter the levels of fasting glucose, glucose tolerance, insulin response or serum insulin levels compared to those of WT mice. In agreement with the unaltered glucose tolerance, the phosphorylation levels of the AKT protein kinase, known to play a central role in regulating insulin-dependent glucose disposal^41,59^, were comparable in skeletal muscles of Tg^sAnk1.5/+^ and WT mice. These results indicate that skeletal muscle-specific overexpression of sAnk1.5, at least in this transgenic mouse model, does not predispose, per se, to a prediabetic or diabetic condition. Alterations in glucose handling in Tg^sAnk1.5/+^ were not even observed following a dietary treatment aiming to promote obesity. Indeed, when Tg^sAnk1.5^ mice were nourished with high fat-containing food, a protocol known to lead to obesity with 60–80% of the resulting weight gain being attributable to body fat^59–62^, the weight gain of WT and Tg^sAnk1.5/+^ mice was comparable, and both glucose tolerance and insulin response were similar in Tg^sAnk1.5/+^ compared to WT mice. This clearly indicates that sAnk1.5 overexpression does not aggravate the obesity outcome or may induce a diabetic phenotype.

On the other hand, we detected a significant increase in caloric intake in Tg^sAnk1.5/+^ mice fed a high-fat diet. This evidence is suggestive of unbalanced calorie intake over energy expenditure in Tg^sAnk1.5/+^ mice. We previously showed that skeletal muscle fibers of mice lacking sAnk1.5 expression displayed altered Ca^2+^ homeostasis^63^. SERCA pumps play a pivotal role in regulating Ca^2+^ homeostasis in skeletal muscle fibers, as they actively drive continuous Ca^2+^ reuptake in the SR, allowing both muscle relaxation and maintenance of adequate SR Ca^2+^ store content. SERCA pump activity thus represents a major ATP-consuming mechanism in skeletal muscle^42,64^. Accordingly, we verified the Ca^2+^-dependent activity of the SERCA pump in muscles from 3- and 14-month-old Tg^sAnk1.5/+^ mice. Interestingly, we observed that V_max_ was increased in muscles from 3-month-old TgsAnk1.5/+ mice but not in those from 14-month-old Tg^sAnk1.5/+^mice. The increased V_max_ values observed in muscles from 3-month-old Tg^sAnk1.5/+^ mice may reflect the higher SERCA1 protein levels in these mice compared to those from age-matched WT mice and thus account for the increased energy demand and calorie intake observed in Tg^sAnk1.5/+^ mice fed a high-fat diet. The increase in K_m_ observed in 14-month-old Tg^sAnk1.5/+^ mice is suggestive of altered SERCA1 regulation in these transgenic mice at this age, including changes in posttranslational modifications or expression of SERCA1 regulators, such as myoregulin, sarcolipin or phospholamban, which are primarily modulators of SERCA1 Ca^2+^ affinity^43–47^. It is worth noting that previous studies reported that sAnk1.5, when expressed in non-muscle cells, is able to regulate the activity of SERCA by physically binding to both SERCA and sarcolipin^34,35^. The results obtained in skeletal muscle of Tg^sAnk1.5/+^ mice, although obtained by an indirect approach, are compatible with this hypothesis, also considering that both sarcolipin and phospholamban expression levels did not differ in muscles of WT and Tg^sAnk1.5/+^ mice. Whether the increase in SERCA K_m_ in observed gastrocnemius muscle from 14-month-old Tg^sAnk1.5/+^ mice may reflect an effect of the overexpression of sAnk1.5 and/or of other regulators of SERCA activity represents an interesting hypothesis to test in the future.

In conclusion, the characterization of glucose homeostasis in Tg^sAnk1.5^ mice indicates that the association of the rs508419 C/C genotype with T2D risk cannot be explained by increased expression of sAnk1.5 proteins in skeletal muscles. At the same time, we noted that increased levels of sAnk1.5 protein in the skeletal muscle of Tg^sAnk1.5^ mice might also affect SERCA activity, which may represent a further function of sAnk1.5 protein, in addition to its established role in stabilizing the SR around the myofibrils.

## Materials and methods

### Mice

All the reported procedures were performed to minimize animal suffering, i.e. isoflurane anesthetized mice were sacrificed by cervical dislocation, as approved by the Animal Care Committee of the University of Siena, and following the approval and authorization of the Italian Ministry of Health (N. 27_2020-PR). All procedures are in compliance with the directive 2010/63/EU of the European Parliament and the Council of 22 September 2010 about welfare of animals used for scientific purposes, and the study is reported in accordance with ARRIVE guidelines (https://arriveguidelines.org). All experiments were performed on adult C57BL/6J male mice (2–14 months of age). Mice had free access to food and water and were housed at room temperature of 21–25 °C and relative humidity of 50–60%, with a dark–light cycle of 12 h. To induce obesity, 2 months old wild type (WT) and Tg^sAnk1.5/+^ male mice on a C57B6/J background were randomly assigned to four experimental groups: control mice (n = 18 WT; n = 18 Tg^sAnk1.5/+^) were fed with a chow standard diet; treated mice (n = 18 WT; n = 20 Tg^sAnk1.5/+^) were fed for 12 weeks with a high-fat diet (HFD) where 45% of metabolized energy was from fats (PF1916, Mucedola s.r.l., Italy).

### Mice generation and genotyping

The murine sAnk1.5 coding sequence, including the untranslated 5′-region, was subcloned into the pMEX vector for skeletal muscle-specific expression^36^ under the control of the muscle-specific rat myosin light chain (MLC) promoter. Microinjection of the pMex-sAnk1.5 DNA into the pronuclei of fertilized one-cell mouse embryos was performed at the Nanjing Biomedical Research Institute of Nanjing University. Transgenic Tg^sAnk1.5/+^ and WT mice were intercrossed to expand both Tg^sAnk1.5/+^ and WT colonies, and the latter (not-carrier sibling mice) was used as a control. Weanlings were genotyped by three independent PCR reactions on genomic DNA extracted from tail tissue with the Gentra Puregene kit (Qiagen, GmbH, Hilden, Germany) according to the manufacturer’s instructions. The three pairs of primers used for mouse genotyping were as follows:

sAnk1.5 Tg-Fw1: 5′-CAAGTGAACCGTCCAATCCA-3′

sAnk1.5 Tg-Rev1: 5′-TGTGGAGATCCAGTTTCTCATTC-3′

sAnk1.5 Tg-Fw2: 5′-CAGATGAACAGGGCAACATTG-3′

sAnk1.5 Tg-Rev2: 5′-TCCCATTCATCAGTTCCATAG-3′

sAnk1.5 Tg-Fw3: 5′-ATAGTGCCTTGACTAGAGATC-3′

sAnk1.5 Tg-Rev3: 5′-GAACCAAAGCATCGACCAGT-3′

Mice were considered transgenic and included in our analysis only if each of the three PCR reactions yielded the expected size of the amplicons.

### Quantitative analysis of sAnk1.5, Sln and Pln mRNA

Skeletal muscles (gastrocnemius, extensor digitorum longus, and soleus) and other tissues (heart, lung, liver, spleen and brain) were dissected from WT and Tg^sAnk1.5/+^ mice. RNA extraction was performed as previously described^65^. cDNAs were obtained by a Promega (Madison, WI) retro-transcription kit and used for quantitative real-time PCR as previously reported^66^, using the following primers: sAnk1.5 Transgene Fw: 5′-GAGGAGATCCTTCTTT TGTTCCA-3′, sAnk1.5 Transgene Rev: 5′-GGACGTGGTGACCCACCTG-3′, Sln For: 5′-GGTGGAGAGACTGAGGTCCTT-3′, Sln Rev: 5′-CCAAGGCTTGTCTTCACTTCCTGA-3′, Pln For: 5′-GATCACCGAAGCCAAGACAGAA-3′, Pln Rev: 5′-CTGGCAAGTTCCTTTGGTCC-3′, GAPDH Fw: 5′-CCAGAATGGGAAGCTTGTC-3′, GAPDH Rev: 5′-TCTCGCTCCTGGAAGATGGT-3′.

The levels of GAPDH enzyme were used for normalization. The rate of expression of the transgene relative to WT was calculated using the comparative Ct method (ΔΔCt)^67^.

### SDS-Page and Immunoblot

Total proteins lysates and microsomes, prepared and quantified as previously described^68,69^, were separated by SDS-Page, in a 10% acrylamide gel. Immunoblotting was performed as in^70^, with minor modifications. Separated proteins were transferred onto a nitrocellulose membrane and stained with Ponceau S (0.2% Ponceau S, .2% Trichloroacetic acid). Membranes were blocked with 5% milk in TBS-T (20 mM Tris–HCl, pH 7.4,150 mM NaCl, and 0.1% Tween 20) for 1 h at room temperature, and then incubated overnight at 4 °C with the primary antibodies diluted in 5% milk in TBS-T. Membranes were incubated for 1 h at room temperature, with HRP-conjugated secondary antibodies (GE Healthcare, UK) diluted 1:3000 in 5% milk in TBS-T solution. For total AKT analysis, p-AKT membrane was stripped at 56 °C for 20 min. in 10 ml of stripping solution (SDS 2%, TRIS–HCl 0.5 M, 2-mercaptoethanol (Sigma-Aldrich, St Louis, MO) 0.08 ml. Washed membranes were incubated with Enhanced Chemi-Luminescence solutions (ECL, Bio-Rad, Hercules, CA, USA) for 5 min and the luminescent signal was acquired by ChemiDoc luminescence counter (Bio-Rad, Hercules, CA, USA). Quantification of the intensities of immunoreactive bands was performed by Image Lab software (Bio-Rad, Hercules, CA, USA), using optical densities of total protein bands pattern as normalizer. The following primary antibodies were used: polyclonal rabbit anti-SERCA TRY2, home-made, used at 1:2000^71^; polyclonal rabbit anti-sAnk1.5, home-made, used at 1:1000^28^; monoclonal anti-calsequestrin-1, clone VIIID12, cat. number MA3-913, Thermo Fisher Scientific (Waltham, MA), used at a 1:1000; polyclonal rabbit anti-AKT(pan), cat. n. 4691, polyclonal rabbit anti-phospho-AKT (Ser473), cat. n. 4060, and polyclonal rabbit anti-phospho-AKT (Thr308), cat. n. 2965, were all from Cell Signaling Technology (Danvers, MA) and used at 1:1000; rabbit anti-Sln, 18395-1-AP was from Proteintech (Rosemont, IL) and used at 1:1000; mouse anti-Pln, MA3-922 was from Thermo Fisher Scientific and used 1:1000.

### Immunofluorescence on isolated EDL fibers

Extensor digitorum longus (EDL) muscles excised from 2-month-old WT and Tg^sAnk1.5/+^ mice were prefixed in 1% PFA for 1 h. Single fibers were mechanically isolated, fixed and permeabilized as previously described^72^. A monoclonal antibody against α-actinin (clone EA-53, Sigma-Aldrich, St Louis, MO, USA) was used to identify the Z-disks, and a polyclonal antibody against sAnk1.5^28^ was used to immunolocalize sAnk1.5. Cy3-conjugated anti-rabbit (Jackson ImmunoResearch, UK) or Alexa Fluor488-conjugated anti-mouse secondary antibodies (Thermo Fisher Scientific) were used for immunofluorescence detection. Fibers were imaged using an LSM510 META confocal microscope with a Plan-Neofluar 20× and 63× 0.50 NA objective and acquired with LSM acquisition software (Carl Zeiss, Jena, Germany).

### Intraperitoneal glucose and insulin tolerance tests

Glucose tolerance tests were performed on 2-, 7-, 10- and 12-month-old mice fed a chow diet and 5-month-old mice following 12 weeks of a high-fat dietetic regimen. Mice were fasted overnight and then weighed. Ten microliters of 20% d-glucose (Sigma-Aldrich) solution per gram of body weight was injected intraperitoneally, corresponding to a final administration of 2 g/kg glucose.

Insulin tolerance tests were performed on 12-month-old mice. After 5 h of daily fasting, the mice were weighed, and 10 μl of insulin solution (100 U/ml diluted 1:1000, Eli Lilly Italia S.p.A.) per gram of body weight was injected intraperitoneally, corresponding to a final administration of 1 U/kg insulin. To measure blood glucose levels, a drop of blood from the tail tip was collected on the glucose strip of a glucometer (OGC care, Biochemical System International, Arezzo, Italy). Blood glucose concentration was measured before the injections (t0); at 30, 60, 120, and 180 min after glucose administration; and at 15, 30, 60, 90 and 150 min after insulin administration. Serum insulin levels were determined by using a mouse insulin ELISA kit with mouse insulin as a standard, following manufacturer’s instructions (Mercodia, Uppsala, Sweden).

### SERCA ATPase activity

Microsomes were prepared from the gastrocnemius muscle of 3- and 14-month-old WT and transgenic mice as previously described^69^. The Ca^2+^-dependent specific ATPase activity of the SERCA1 pump was determined in microsomal preparations as described in^73^. The rate of ATP hydrolysis catalyzed by the microsomal membranes was determined at 37 °C in 96-well plate format through the release of inorganic phosphate (Pi) over a period of 20 min using the Baginski method^74^. Briefly, the reaction mix containing 50 mM TES/TRIS, pH 6.9, 100 mM KCl, 7 mM MgCl_2_, 1 mM ethylene glycol‐bis(β‐aminoethyl ether)‐N,N,N′,N′‐tetraacetic acid (EGTA), and 250 ng of microsomes, as well as CaCl_2_ at different concentrations, was initiated with 5 mM ATP and incubated at 37 °C for 20 min. The hydrolysis of ATP was stopped by adding ice-cold ascorbic acid solution (170 mM ascorbic acid dissolved in 0.5 N HCl and mixed with 4 mM ammonium heptamolybdate) and by gradually removing the wells from the heating plate. Subsequently, the plate was placed on ice for at least 5 min, and the colorimetric solution (150 mM sodium-m-arsenite, 70 mM sodium citrate, 0.35 mM acetic acid) was transferred to each well. The plate was kept at 37 °C for 5 min to complete the reaction. Absorbance was evaluated at 850 nm wavelength, and measurements obtained were related to a standard series of known Pi concentrations treated in parallel during the ATPase assay. The results were analyzed with Excel and Prism (GraphPad Software, San Diego, CA, USA) software. The rate of ATP hydrolysis was also normalized to SERCA1 relative expression levels obtained by Western blot experiments performed on the same microsome preparations used to perform the ATPase assay. The relative specific ATPase activity (rate of ATP hydrolysis expressed as nmol Pi/% SERCA/min) was normalized to the maximal SERCA1 activity of the age-matched WT mice, calculated at each free [Ca^2+^], and plotted as a function of the free [Ca^2+^]. The kinetic parameters (V_max_, K_m_, n) were derived from nonlinear regression analysis, following dose–response model fitting of the rising part of each curve. The parameters generated by the dose–response function Top, EC50, and Hill slope correspond to V_max_, K_m_, and n, respectively.

### Statistical analysis

Data are expressed as the mean ± standard deviation (SD). Comparisons between experimental groups for each variable investigated were carried out with unpaired Student’s t-test. Areas under the curve (AUCs) obtained following glucose or insulin tolerance tests were compared by two-way ANOVA, followed by Bonferroni’s post hoc test. An F-test was used to test the significance, and a p value of less than 0.05 was considered statistically significant. All statistical analyses were performed with Prism software.

## Supplementary Information

Supplementary Figures.

## Data Availability

All data generated during this study are included in this published article.
