# Supplementary material for: Skeletal muscle overexpression of sAnk1.5 in transgenic mice does not predispose to type 2 diabetes
Source: Sci Rep. 2023 May 20;13:8195. doi: 10.1038/s41598-023-35393-0 (PMC10199891; doi:10.1038/s41598-023-35393-0)
Supplement: Supplementary file 1 — Supplementary Figures. [file 41598_2023_35393_MOESM1_ESM.pdf]

Skeletal muscle overexpression of sAnk1.5 in transgenic mice does not predispose to type 2 diabetes

Pierantozzi E[1*], Raucci L[1*], Buonocore S[1], Rubino EM[1], Ding Q[2], Laurino A[1], Fiore F[1], Soldaini M[1], Chen J[4], Rossi D[1,3], Vangheluwe P[4], Chen H[2,5], Sorrentino V[1,3#].

[1]Department of Molecular and Developmental Medicine, University of Siena, 53100, Siena, ITALY,
[2]Department of Cardio-Thoracic Surgery, Nanjing Drum Tower Hospital, Nanjing University Medical School, Nanjing, 210008, Jiangsu, China
[3]Interdepartmental Program of Molecular Diagnosis and Pathogenetic Mechanisms of Rare Genetic Diseases, Azienda Ospedaliero Universitaria Senese, 53100, Siena, Italy.
[4] Laboratory of Cellular Transport Systems, Department of Cellular and Molecular Medicine, Katholieke Universiteit Leuven (KU Leuven), 3000 Leuven, Belgium.
[5] Programme in Cardiovascular and Metabolic Disorders, Duke-NUS Medical School, 8 College Road, 169857 Singapore.

Supplementary figures

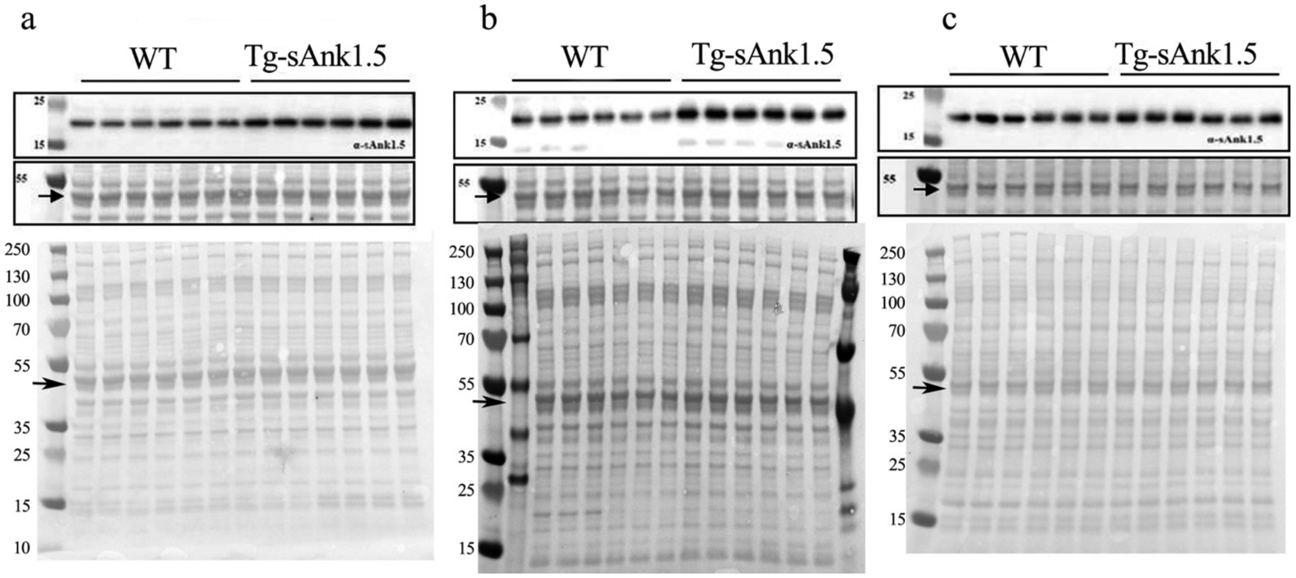

Supplementary Figure S1: Upper panels report western blots of sAnk1.5 expression levels in gastrocnemius (a), EDL (b), and soleus (c) from WT and Tg$^{sAnk1.5/+}$ mice, as in Figure 3a, 3b and 3c. Lower panels reports the corresponding original full membranes stained with ponceau S. The arrow points to the gel region corresponding to actin used as a normalizer of gel loading.

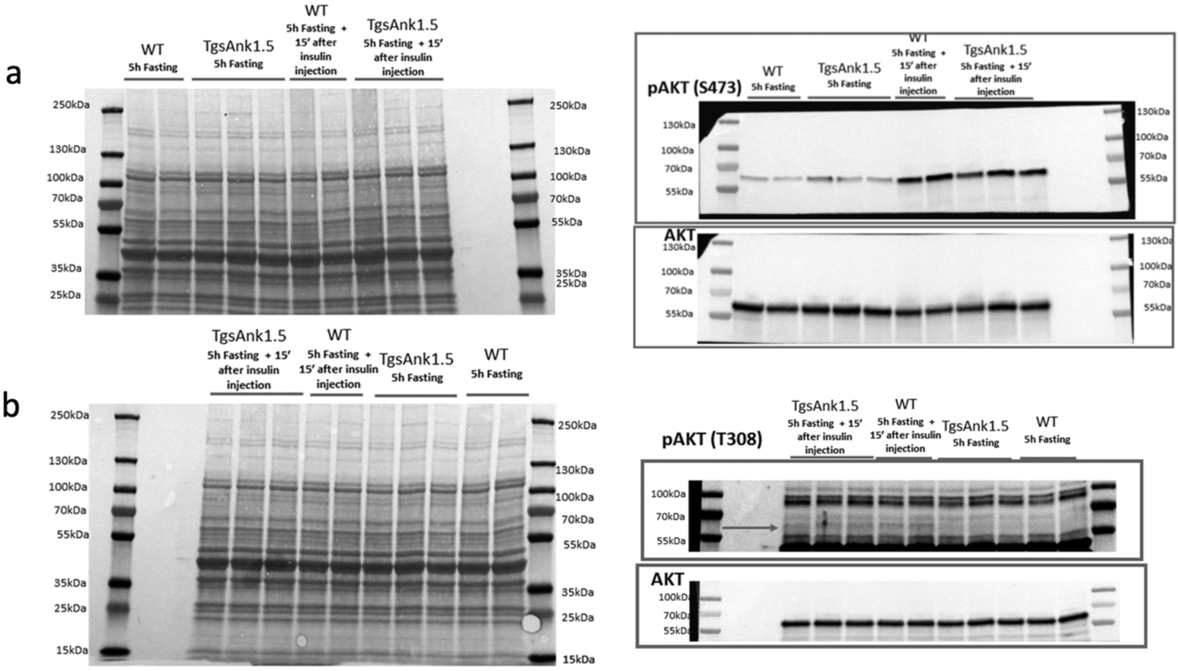

Supplementary Figure S2: AKT phosphorylation levels following fasting and insulin administration.

a: right panels report western blots of pAKTSer473 and of total AKT expression levels in gastrocnemius muscle from WT and $Tg^{sAnk1.5/+}$ mice, as reported in Figure 4f. Left panel reports the corresponding original full membrane stained with ponceau S.

b: right panels report western blots of pAKT-Thr308 and of total AKT expression levels in gastrocnemius muscle from WT and $Tg^{sAnk1.5/+}$mice. Left panel reports the corresponding original full membrane stained with ponceau S. The arrow points to the band corresponding to the pAKT-Thr308.

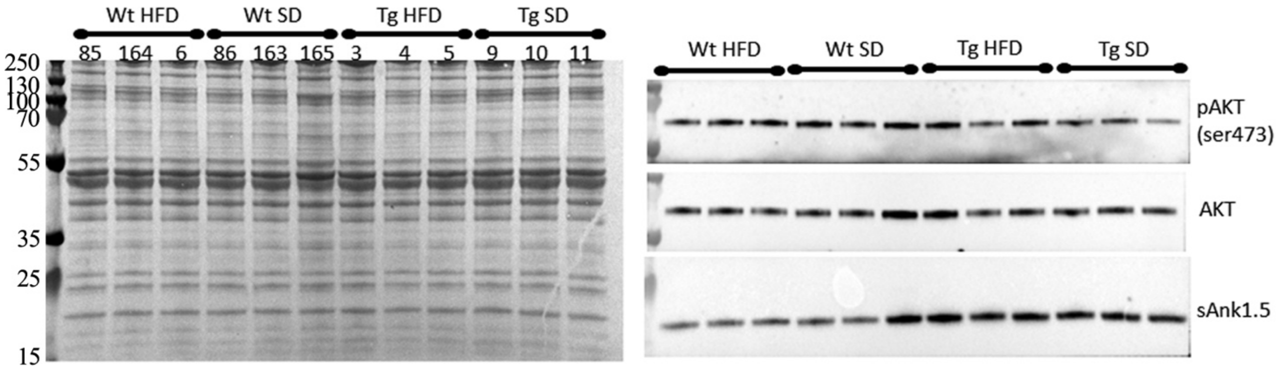

Supplementary Figure S3: Original blots/gel of pAKTSer473 expression levels in chow- and high fat fed WT and $Tg^{sAnk1.5/+}$ mice.

Right panels report western blots of pAKTSer473, total AKT and sAnk1.5 expression levels in gastrocnemius muscle from WT and $Tg^{sAnk1.5/+}$ mice fed with a chow (standard diet, SD) or a high fat diet (HFD), as reported in Figure 5g. Left panel reports the corresponding original full membrane stained with ponceau S. Mouse identification tag is indicated by the number on top of each lane.

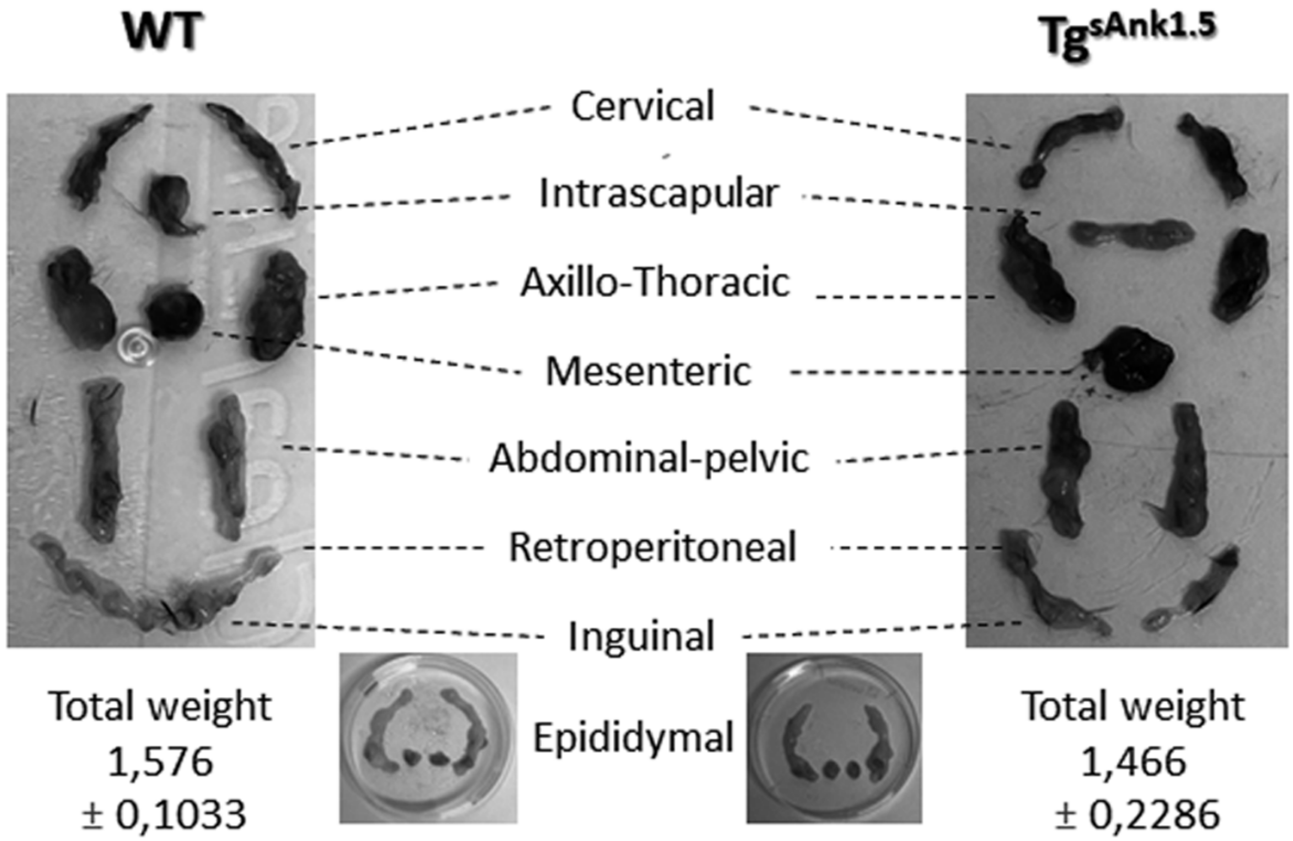

Supplementary Figure S4: Anatomical distribution of adipose tissue dissected from WT and $Tg^{sAnk1.5/+}$ mice.

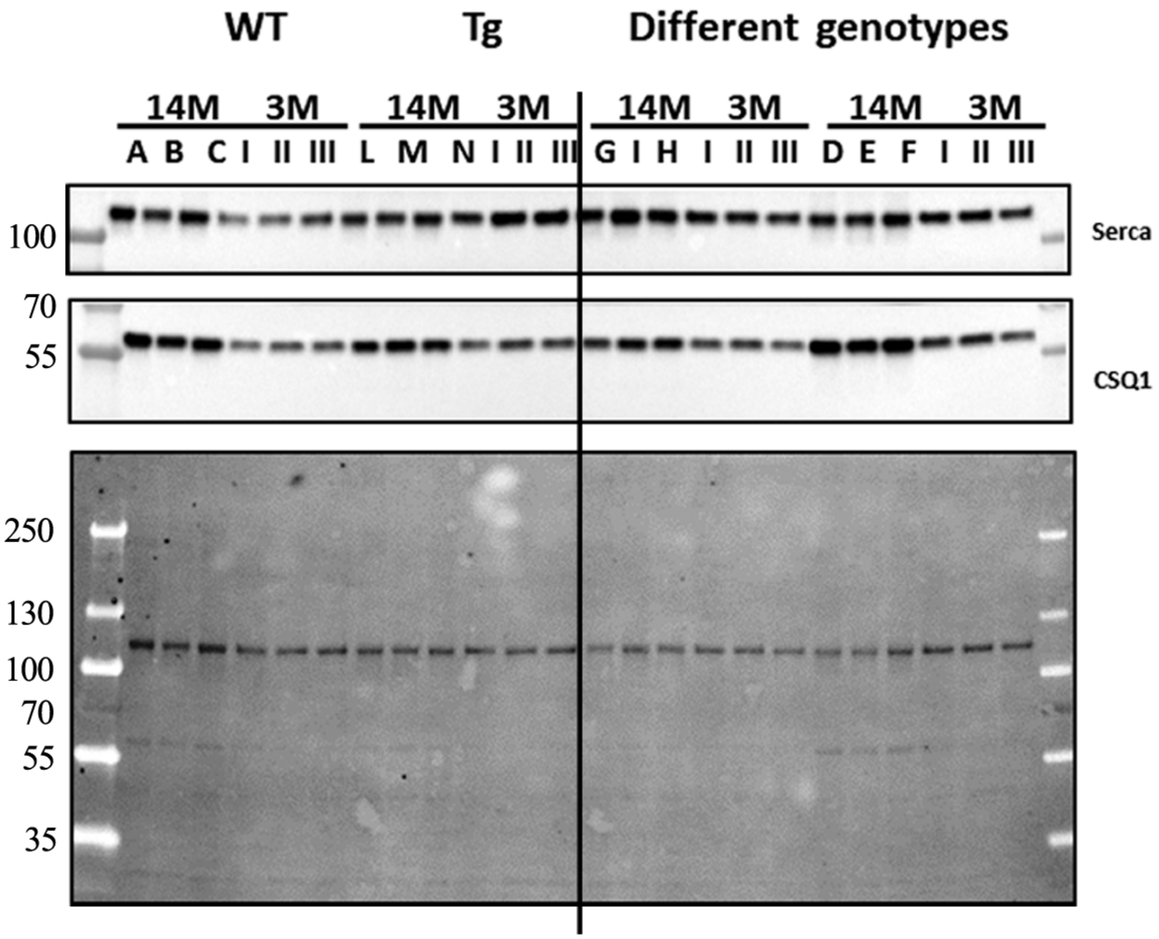

Supplementary Figure S5: SERCA expression levels in microsome preparations from gastrocnemius muscle. The upper panel report western blots of SERCA and calsequestrin (CSQ1) expression levels in gastrocnemius muscle from WT and $Tg^{sAnk1.5/+}$ mice at 3 and 14 months of age as reported in Figure 7c. Lower panel reports the “stain-free imaging” of the corresponding gel. Mouse tag is indicated by the letters/roman numbers on top of each lane. The left part of the image was cropped and reported in Fig. 7c.

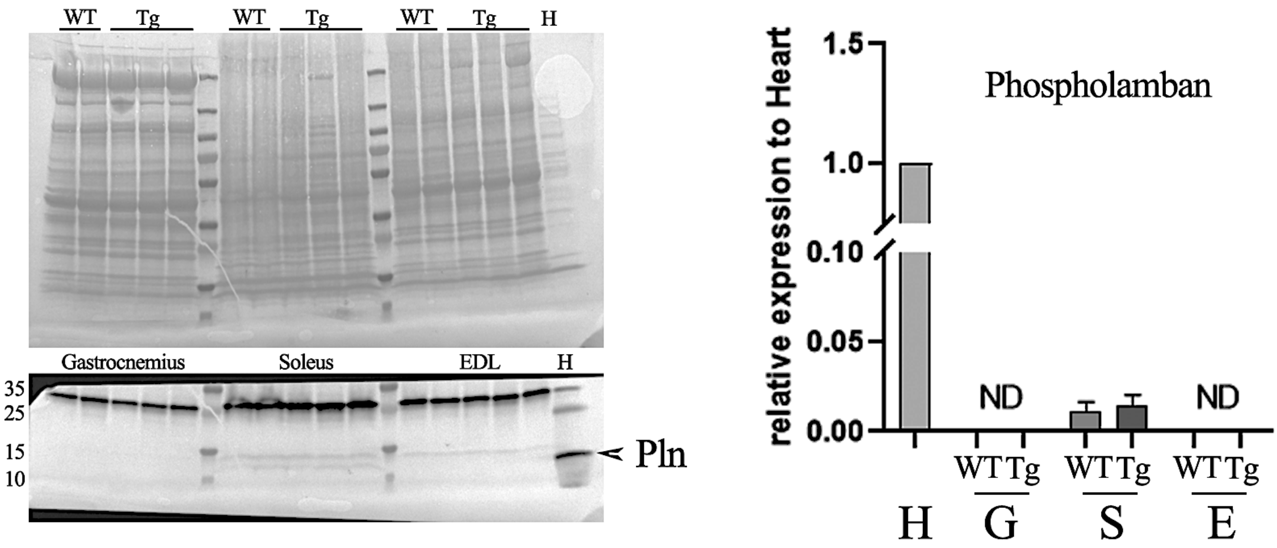

Supplementary Figure S6: Phospholamban expression levels in gastrocnemius (G), Soleus (S), EDL muscles and heart (H) from WT and Tg$^{sAnk1.5/+}$ mice. The left upper panel reports original membrane stained with ponceau S of the corresponding western blot stained with antibodies against Phospholamban (Pln). The right panel reports the relative quantification of Pln expression from the same western blot.
